# Use of High- and Medium-Cut-off Membrane Hemodialysis for Removal of Free Light Chains in Patients with Multiple Myeloma—A Single-Center Experience

**DOI:** 10.3390/jcm15082917

**Published:** 2026-04-11

**Authors:** Matevz Skerget, Barbara Vajdic Trampuz, Tajda Starman, Jakob Gubensek

**Affiliations:** 1 Department of Hematology, University Medical Center Ljubljana, Zaloska Cesta 2, 1000 Ljubljana, Slovenia; tajda.starman@kclj.si; 2 Faculty of Medicine, University of Ljubljana, Vrazov Trg 2, 1000 Ljubljana, Slovenia; barbara.vajdic.trampuz@kclj.si (B.V.T.); jakob.gubensek@kclj.si (J.G.); 3 Deparment of Nephrology, University Medical Center Ljubljana, Zaloska Cesta 2, 1000 Ljubljana, Slovenia

**Keywords:** acute kidney injury, myeloma, light-chain cast nephropathy, high-cut-off dialysis, medium-cut-off dialysis, free light chains

## Abstract

**Background/Objectives**: Light-chain cast nephropathy remains a major cause of morbidity in newly diagnosed multiple myeloma (MM), and rapidly reducing circulating free light chains (FLCs) is considered essential for renal recovery and survival. **Methods**: We conducted a single-center retrospective study evaluating high- and medium-cut-off hemodialysis (HCO/MCO HD) in newly diagnosed MM patients presenting with acute kidney injury (AKI). Consecutive patients treated at the University Medical Center Ljubljana between 1 January 2020 and 31 December 2023 were included. As per institutional protocols, HCO/MCO HD- and myeloma-directed therapy were initiated on the day of diagnosis. Primary endpoints were the magnitude of FLC reduction, renal and hematologic responses at three months, and overall survival. **Results**: The median FLC concentration at presentation was 9630 mg/L. FLC levels declined rapidly after HCO/MCO HD initiation, reaching 2400 mg/L by day 7, 1083 mg/L by day 14, and 370 mg/L by day 30. MCO HD achieved kapa FLC clearance comparable to HCO HD for the lambda isotype. Despite a median of only four HCO/MCO-HD sessions, the reduction in FLC was rapid, with an additional decline observed over time, while the median FLC concentration fell below 500 mg/L. At three months, the overall hematologic response was 87%, including very good partial response or better in 35% of patients, and renal response in 79% of patients. Achieving a ≥70% FLC reduction by day 7 was associated with superior outcomes, including markedly longer median overall survival (82.5 vs. 23.2 months). **Conclusions**: Our data show that HCO/MCO-HD treatment alongside anti-myeloma therapy achieves sustained FLC reduction in newly diagnosed MM with AKI and early FLC reduction is highlighted as a key determinant of survival.

## 1. Introduction

Acute kidney injury (AKI) caused by light-chain cast nephropathy (LCCN) is a common complication in patients with newly diagnosed multiple myeloma (MM). The incidence of AKI in this context reaches up to 50%, with approximately 10% of patients requiring dialysis [1,2,3,4]. Myeloma cells overproduce free light chains (FLCs), which are then filtered through the glomerular membrane. Interaction of FLCs with uromodulin in the loop of Henle leads to the formation of casts and obstruction of tubules. Additionally, increased reabsorption of FLC activates NF-κB and other signaling pathways (ASK1 and STATs), leading to apoptosis, inflammation and tubulointerstitial fibrosis [4,5].

Kidney injury in MM is defined by an estimated glomerular filtration rate (eGFR) of less than 40 mL/min/1.73 m^2^ [6]. Before attributing AKI to LCCN, other potential causes, such as dehydration, hypercalcemia, and monoclonal gammopathy of renal significance (MGRS), should be ruled out [4]. Kidney biopsy is the gold standard for distinguishing between LCCN, MGRS-related lesions, and other unrelated causes of AKI. However, in acute situations, a biopsy may not always be feasible. A substantial FLC burden, with concentrations surpassing 500 mg/L and marked FLC proteinuria, is typically indicative of LCCN [4]. In contrast to other renal conditions such as amyloidosis and MGRS, the ratio of albuminuria to total proteinuria tends to be low. Therefore, in patients with more than 1 g/day of proteinuria and less than 10% albuminuria, combined with a serum FLC concentration exceeding 1500 mg/L, the likelihood of LCCN is high enough that a kidney biopsy may not be necessary [4,6,7]. Clinical expertise is crucial for differentiating and excluding other causes of AKI.

In MM patients, AKI is associated with lower overall survival and increased mortality, while improvement in renal function is associated with better survival [1,7,8]. Therefore, prompt treatment is crucial to reduce FLC levels. Kappa and lambda FLCs have a monomeric molecular weight of approximately 22.5–25 kDa. In human serum, kappa FLCs predominantly circulate as monomers (~22.5 kDa), and lambda FLCs are mainly present as covalently linked dimers with an approximate molecular weight of 45 kDa [4]. Extracorporeal FLC elimination using hemodialysis-based techniques has been employed to enhance FLC removal in patients with LCCN. Due to their larger molecular size, lambda FLCs are typically removed using high-cut-off (HCO) dialyzers, whereas kappa FLCs can be effectively cleared using medium cut-off membranes (MCO) [4,9]. The use of MCO membranes enables clearance of medium- and large-molecular-weight solutes comparable to HCO membranes, while minimizing albumin loss [9,10]. These properties make MCO membranes a potentially safer and more cost-effective adjunctive therapy for the treatment of LCCN.

High- and medium-cut-off membrane hemodialysis (HCO/MCO-HD) enhances FLC clearance, but evidence from clinical studies remains mixed and has not consistently demonstrated improved renal outcomes and survival [11,12,13,14,15,16]. In the MYRE study, the use of HCO-HD led to an overall response rate (ORR) of 78.3% compared to 60.4% with standard hemodialysis (*p* = 0.06) and a higher rate showing a very good partial response (VGPR) or better (69.6% vs. 47.9%, *p* = 0.03) at 6 months [11]. HCO-HD also resulted in greater renal recovery at both 6 months (56.4% vs. 35.4%, *p* = 0.04) and 12 months (60.9% vs. 37.5%, *p* = 0.02). However, there was no significant difference in overall survival between the two groups [11]. Conversely, the EuLITE study found that patients receiving HCO-HD had a lower response rate compared to those receiving standard hemodialysis, with complete response (CR) rates of 14% versus 30% and VGPR rates of 23% versus 32%. Additionally, the HCO-HD group had worse survival outcomes, possibly due to a higher incidence of infectious complications [12].

In the aforementioned randomized studies, myeloma-specific treatment consisted of either a doublet regimen with bortezomib and dexamethasone or a triplet regimen that included doxorubicin alongside bortezomib and dexamethasone in the MYRE and EuLITE studies, respectively [11,12]. These regimens are not currently considered standard therapy, as most patients now receive either a triplet incorporating bortezomib and lenalidomide, or a quadruplet including an anti-CD38 antibody [7]. Both studies employed an aggressive HCO-HD protocol at initiation, which likely resulted in the removal of other plasma proteins and may have contributed to the elevated infection rates and treatment interruptions in the EuLITE study [12]. Furthermore, the MYRE study incorporated a screening period of 4–15 days prior to HCO-HD, and this delay in treatment initiation may have reduced the response rate due to ongoing AKI.

There is currently no data from randomized studies evaluating the efficacy of HCO/MCO-HD in patients undergoing treatment with novel induction combinations. Furthermore, key randomized trials that led to the approval of daratumumab, such as Cassiopeia, Griffin, and Perseus studies, excluded patients with an eGFR of less than 30 mL/min/1.73 m^2^ [17,18,19]. In a limited cohort of 20 patients with AKI, of whom 9 required hemodialysis at the time of presentation, induction therapy based on daratumumab resulted in dialysis independence rates of 57.1% at 3 months and 85.7% at 12 months [20].

More research is needed on the impact of HCO/MCO-HD in newly diagnosed MM with AKI, particularly with new therapies emerging. Our center’s protocol entails the immediate initiation of induction therapy and daily HCO/MCO-HD in newly diagnosed MM patients presenting with AKI in the setting of markedly elevated FLC levels and an eGFR below 15 mL/min/1.73 m^2^, with adjustments to the procedure based on recurrent monitoring of FLC levels.

Given the considerable cost, potential adverse events associated with this procedure, and limited evidence supporting its efficacy, we conducted an analysis of the outcomes of our approach.

## 2. Materials and Methods

This retrospective study enrolled newly diagnosed MM patients, presenting with AKI requiring dialysis at the University Medical Center Ljubljana between 1 January 2020 and 31 December 2023. The initiation of HCO/MCO-HD was determined by the attending nephrologist, in consultation with a hematologist, when patients exhibited new-onset acute kidney injury without a prior history of severe chronic kidney disease and demonstrated elevated free light chains exceeding 1000 mg/L. Electronic patient records were reviewed for genetic and biochemical data at diagnosis, time from diagnosis to starting myeloma-specific therapy, type of myeloma-specific therapy, and time to and number of HCO/MCO-HD procedures. Patients with established advanced kidney disease (CKD stages 4 and 5) were excluded, as our protocol does not involve initiating HCO/MCO-HD immediately in these individuals. Longitudinal data for FLC reduction on days 7, 14, and 30 post-HCO/MCO-HD were collected. Renal and hematological responses followed IMWG criteria [6,21]. MM was stratified by RISS and cytogenetic risk using the 2025 IMS–IMWG system [22]. The main endpoints were renal and hematological responses at 3 months and overall survival.

The study protocol was reviewed and approved by the national ethics committee (KME 0120-8/2025-2711-3). The research was conducted in accordance with the ethical standards of the Declaration of Helsinki. Given the retrospective nature of the study, adherence to local laws, and the use of anonymized data, the requirement for informed consent was waived.

### 2.1. Dialysis Procedure

In patients with MM and predominant lambda, FLC intermittent dialysis using a high-cut-off (HCO) membrane (Theralite^®^; Gambro Dialysatoren, Hechingen, Germany) was performed in hemodiafiltration mode. Treatments were delivered with a blood flow rate of 250–300 mL/min and post-dilution replacement fluid at 3 L/h, with a typical session duration of 6–8 h. According to our institutional HCO dialysis protocol, patients routinely received 200 mL of 20% human albumin during the final phase of each procedure [23]. In patients with MM and predominant kappa FLCs, intermittent hemodialysis using a medium-cut-off (MCO) membrane (Theranova^®^; Gambro Dialysatoren, Hechingen, Germany) was performed with a blood flow rate of 250–300 mL/min for a duration of 4–6 h per session, without albumin supplementation. Hemodialysis sessions were performed daily or on alternate days, depending primarily on circulating FLC levels.

### 2.2. Statistical Analysis

Descriptive statistics were used to summarize patient characteristics. Normality was assessed using the Shapiro–Wilk test, and data were described using medians with Q1 and Q3 quartiles or means with standard deviations, as appropriate. Median free-light-chain (FLC) concentrations at each timepoint were estimated together with 95% non-parametric bootstrap confidence intervals, and paired differences between timepoints were evaluated using the Wilcoxon signed-rank test. Associations between predictors and the probability of achieving renal and hematologic responses were assessed using Firth’s penalized logistic regression to reduce small-sample bias and address potential separation. Overall survival (OS) was defined as the time from diagnosis—established as the date of bone marrow biopsy—to death from any cause; patients alive at last follow-up were censored. OS was estimated using the Kaplan–Meier method, with subgroup comparisons performed using the log-rank test. Univariate associations with OS were examined using Cox proportional hazards models; multivariable analysis was not undertaken due to an insufficient number of events for reliable model estimation. Median follow-up was calculated using the reverse Kaplan–Meier method. All statistical tests were two-tailed with a significance level of 0.05. Analyses were performed using RStudio 2026.01.0 and R version 4.5.2.

## 3. Results

### 3.1. Patient Characteristics

A total of 33 patients with newly diagnosed MM were included in the study. The median age was 66 years, with 36% of patients older than 70. There was a slight predominance of male patients. Most participants (64%) had light-chain myeloma (Bence Jones), while the remaining cases were classified as IgG or IgA myeloma. Thirty-three percent of patients exhibited cytogenetically high-risk disease according to the 2025 IMS–IMWG and 42% were classified as RISS stage III. Eighty percent of patients received bortezomib-based induction therapy, and 18% of patients were treated with daratumumab-based regimens that contained bortezomib. In most cases, both myeloma-specific therapy and HCO/MCO-HD were initiated on the same day as the bone marrow biopsy, with the median interval from diagnosis to the start of either treatment or initiation of HCO/MCO-HD being zero days. The estimated median follow-up was 62 months. Table 1 presents patient characteristics.

### 3.2. Free-Light-Chain Assessment

**Figure 1:**
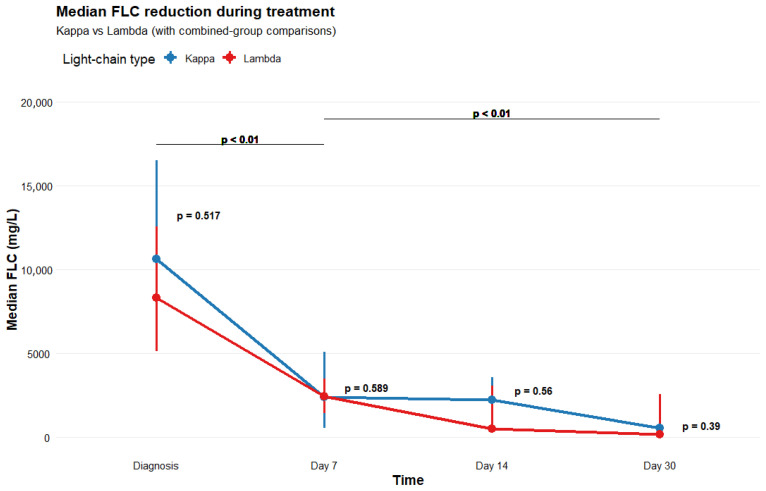
Free-light-chain concentrations during the first 30 days of treatment (median with Q1–Q3 intervals).

FLC levels at diagnosis were markedly elevated, with a median of 9630 mg/L (Q1–Q3 5220–16,000 mg/L). Following initiation of HCO/MCOHD, FLC concentrations declined rapidly, reaching median values of 2400 mg/L (Q1–Q3 575–3650 mg/L) by day 7, 1083 mg/L (Q1–Q3 384–3607 mg/L) by day 14, and 370 mg/L (Q1–Q3 29–2235 mg/L) by day 30. There were no significant differences between kappa and lambda FLC levels at any time, showing that MCO-HD removed kappa FLCs as effectively as HCO-HD for lambda FLC. Despite patients receiving a median of only four HCO/MCOHD sessions, FLC levels continued to fall over time, with significantly lower concentrations on day 30 compared with day 7 (*p* < 0.01) for both light-chain types. Figure 1 depicts the longitudinal trajectory of median FLC concentrations, including values for comparisons between kappa and lambda at each timepoint, as well as paired analyses from diagnosis to day 7 and from day 7 to day 30 for the combined cohort.

### 3.3. Response Assessment

At 3 months, hematological OR and ≥VGPR were 87% and 35%, respectively. Renal response at 3 months was achieved in 79% of patients, including 32% with CR, 43% with PR, and 4% with MR. During the first 3 months of treatment, four patients died. Of these, three were older than 80 years. Two deaths were attributed to progressive disease, one resulted from infectious complications, and one was caused by acute myocardial infarction with subsequent heart failure.

**Figure 2:**
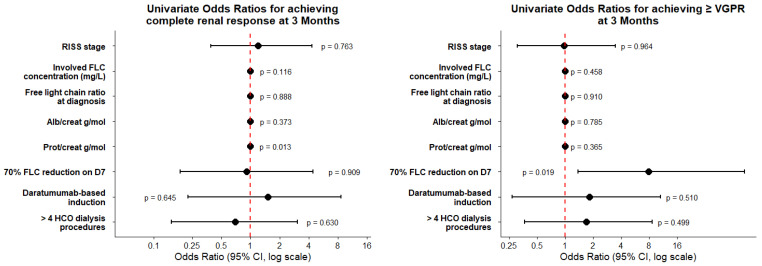
Univariate odds ratios for achieving complete renal response or ≥VGPR at 3 months.

The RISS stage involved the FLC concentration and FLC ratio at time of diagnosis, and was not associated with probability of achieving ≥VGPR or a complete renal response at 3 months. Patients receiving daratumumab-based induction and those undergoing more than four HCO/MCO-HD procedures demonstrated numerically higher rates of complete renal response and ≥VGPR. However, the corresponding confidence intervals were wide, indicating substantial imprecision. Given the limited sample size, this study was unable to detect modest differences, and these findings should be interpreted with caution (Figure 2).

We further examined the predictive power of the albumin/creatinine and protein/creatinine ratio in urine, the concentration of uninvolved FLCs, and early FLC responses on achieving renal recovery or hematologic remission. No association was observed between urine albumin/creatinine or protein/creatinine ratios and response to treatment. There was a statistically significant association of an early decrease in FLC and hematological response. A reduction in FLC by 70% at day 7 was associated with increased odds of achieving ≥VGPR (OR = 7, *p* = 0.019). No statistically significant association was observed between early free-light-chain (FLC) response and renal recovery (Figure 2).

### 3.4. Overall Survival

**Figure 3:**
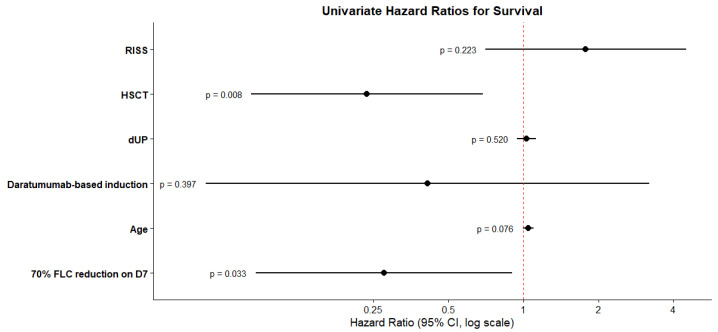
Hazard ratios for OS.

**Figure 4:**
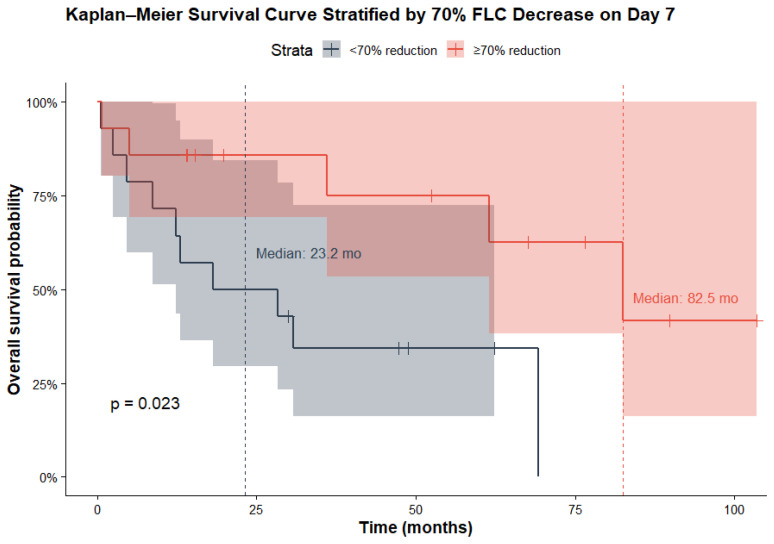
Kaplan–Meier survival curves for OS stratified by early FLC response on day 7.

We subsequently evaluated several factors as potential predictors of overall survival, including daratumumab induction therapy, hematopoietic stem cell transplantation (HSCT), RISS myeloma stage, patient age, and early response in free-light-chain (FLC) reduction. Among these variables, only receiving HSCT and achieving at least a 70% reduction in FLC levels by day 7 were associated with a statistically significant improvement in overall survival, while age had borderline significance. Specifically, HSCT was associated with a hazard ratio (HR) of 0.24 (*p* = 0.008), and a 70% reduction in FLC by day 7 corresponded to an HR of 0.28 (*p* = 0.03), indicating a substantially lower risk of mortality for patients meeting these criteria (Figure 3). Figure 4 presents Kaplan–Meier survival curves comparing patients based on whether they achieved at least a 70% reduction in free-light-chain (FLC) levels by day 7. The median overall survival (OS) was significantly longer in patients who achieved this reduction, with a median OS of 82.5 months compared to 23.2 months in those who did not (log-rank test, *p* = 0.023).

## 4. Discussion

In newly diagnosed MM, acute kidney injury requiring hemodialysis is associated with reduced overall survival and higher early mortality [1,7,8]. Rapid initiation of anti-myeloma therapy is essential, whereas evidence from randomized trials on HCO-HD remains conflicting, partly due to heterogeneous study designs and differing treatment approaches. In this study, we present results from our retrospective cohort, where anti-myeloma and HCO/MCO-HD treatment was initiated simultaneously, and HCO/MCO-HD was tailored to FLC class and levels.

HCO/MCO-HD removes only the intravascular fraction of FLCs, which represents approximately 12–20% of the total FLC burden. Because production is continuous and redistribution from the extravascular compartment occurs, the risk of biochemical rebound and ongoing AKI persists. Novel agents, through their rapid cytoreductive activity, suppress FLC production more effectively and more rapidly than historically used regimens. In our cohort, median FLC concentrations declined markedly within the first 7 days. Consistent with the results reported by Veldman et al., a comparable decrease in both kappa and lambda FLC was observed [16]. MCO-HD demonstrated comparable efficacy in removing kappa FLC to that of HCO-HD in eliminating lambda FLC, while MCO membranes offer superior cost-effectiveness and safety. Despite the limited number of HCO/MCO-HD sessions, FLC levels remained suppressed and continued to decrease between days 7 and 30. These observations suggest that early simultaneous initiation of anti-myeloma therapy with novel agents and HCO/MCO-HD effectively controls disease activity, leading to reduced FLC secretion; a prolonged course of HCO/MCO-HD is unlikely to be necessary, as no rebound in FLC concentrations was detected. Our limited-procedure approach was effective and compared favorably with the results reported in the MYRE study [11]. By day 30, our cohort reached a median FLC level of 370 mg/L, whereas only 43.5% of patients in the Myre study achieved an FLC concentration below 500 mg/L by the end of the first cycle—a threshold considered protective against further kidney injury.

Acknowledging the limitations inherent to a retrospective design and the advanced age of our cohort, we focused our analysis on early mortality within the first 3 months of treatment. Among the four deaths observed, three occurred in patients older than 80 years, and only one was attributed to infectious complications. Although the sample size is limited, these observations may indicate that a shorter, more restricted HCO/MCO-HD strategy could represent a reasonable and potentially safer alternative to prolonged treatment.

At the 3-month evaluation, 87% of patients achieved an ORR, and 35% achieved a ≥VGPR. In comparison, the MYRE study reported a 3-month ORR of 89.1% and a ≥VGPR rate of 60.9%, while the EuLITE study reported 6-month response rates with an ORR of 63% and ≥VGPR of 37% [11,12]. These differences must be interpreted in the context of substantial variation in patient populations across studies. In the MYRE cohort, only 15% of patients in the HCO-HD arm had high-risk disease according to the older risk-stratification system, whereas in our cohort, 33% of patients had high-risk disease based on the newer and more stringent 2025 IMS–IMWG criteria [11]. Despite these differences, our results indicate lower efficacy compared to patients without AKI, particularly regarding the achievement of ≥VGPR, when compared with historical data. With VRd induction, approximately 55% of patients typically achieve a ≥VGPR at 3 months, and ≥VGPR rates of around 80% have been reported, with modern quadruplet regimens incorporating daratumumab after early induction.

Despite achieving only modest hematologic responses, our cohort demonstrated a renal response rate of 79%, including 32% complete renal responses. This compares favorably with the reported independence of approximately 60% of patients from dialysis in the MYRE and EuLITE studies [11,12]. At the same time, our cohort differed in several ways from earlier study populations. First, induction therapy and HCO/MCO-HD were initiated immediately in our cohort, whereas the median time to induction treatment in the Myre study was 8 days. Second, in the EuLITE study, 74% of patients received conventional dialysis prior to starting the treatment protocol, while our patients proceeded directly to HCO/MCO-HD. These distinctions suggest that our population may have had less established AKI at baseline, which is consistent with the hypothesis that early initiation of induction therapy combined with HCO/MCO-HD may facilitate more rapid and complete renal recovery.

Our findings also compare favorably with studies that did not incorporate HCO-HD. In a prospective study evaluating daratumumab and dexamethasone in 38 patients with relapsed or refractory multiple myeloma, 17 of these patients were dependent on dialysis; their overall response and VGPR rates were 47% and 29.4%, respectively, with only one patient (5.9%) achieving a renal response [24]. Among the 21 patients who did require dialysis, the renal response rate was 28.6% [24]. In another retrospective study of 20 newly diagnosed patients, including 9 who required dialysis, 57.1% and 85.7% of dialysis-dependent patients achieved dialysis independence at 3 and 12 months, respectively [20].

We found no association between baseline characteristics at diagnosis and the probability of achieving ≥VGPR or a complete renal response at 3 months. Neither the RISS risk stratification nor the absolute FLC concentration at diagnosis, nor the involved/uninvolved FLC ratio, showed any association with the likelihood of achieving a hematologic or renal response. The urine albumin/creatinine and protein/creatinine ratios were evaluated as markers of albuminuria and potential indicators of kidney injury beyond LCCN. The median albumin/creatinine ratio in our cohort was low (25 g/mol) despite substantial daily proteinuria (median 3.6 g/day), suggesting a predominance of overflow light-chain proteinuria consistent with LCCN. Although the ORs were close to one, indicating no association, the predominance of low albumin/creatinine ratios in our cohort limits the ability to fully assess the predictive value of this parameter for the probability of achieving renal or hematologic response to HCO-HD.

Treatment characteristics, including the use of daratumumab-based induction and a higher number of HCO-HD procedures, were not associated with the probability of achieving ≥VGPR or a complete renal response at 3 months. An early FLC reduction by day 7 was predictive of achieving a better hematologic response at 3 months, likely reflecting a subgroup with more chemosensitive disease compared with patients who exhibited a slower decline. Furthermore, an early FLC response was prognostic for longer overall survival, with a median OS of 82.5 months in patients who achieved a ≥70% reduction by day 7 compared with 23.2 months in those who did not. However, this early FLC response did not translate into a higher probability of renal response at 3 months. These findings are consistent with the MYRE and EuLITE trials, both of which showed no difference in dialysis independence at 3 months [11,12]. Although the MYRE trial reported higher dialysis independence at 6 and 12 months, these were secondary endpoints and should be interpreted as exploratory findings. In contrast, the EuLITE study demonstrated no improvement in dialysis independence, likely influenced by higher infectious complications and treatment interruptions associated with the intensive HCO-HD protocol used in this study.

A major limitation of this study is its retrospective, single-cohort design, which introduces inherent bias, together with a relatively small sample size that limits the statistical power for subgroup analyses. As a result, we cannot fully evaluate the importance of daratumumab-based induction in patients treated with HCO/MCO-HD. As our study included only patients receiving HCO/MCO-HD, it did not allow for a comparison with standard high-flux dialysis. Additionally, all patients were treated according to our institutional protocol with immediate initiation of anti-myeloma therapy and HCO/MCO-HD. Thus, the available data do not allow us to determine whether HCO/MCO-HD provides any incremental benefit over induction therapy with novel agents alone, nor can we conclude that any synergistic effect exists between these approaches. Although our results compare favorably with small studies employing novel agents without HCO-HD, cross-study comparisons are inherently problematic [20,24]. Comparisons with published studies must be interpreted with caution. Existing cohorts differ substantially in terms of disease stage, dialysis dependence, timing of HCO/MCO-HD initiation, and the use of contemporary agents such as daratumumab, proteasome inhibitors, or IMiDs. Lastly, the initiation of HCO/MCO-HD in our cohort was guided solely by patient history and biochemical markers—specifically, elevated FLC levels and the type of proteinuria—rather than kidney biopsy. Acute kidney injury in MM is usually multifactorial, arising from LCCN, dehydration, light-chain amyloidosis, and drug toxicity.

Our data suggest that early initiation of anti-myeloma treatment and a time-limited HCO/MCO-HD strategy can achieve encouraging renal response rates while maintaining low rates of infectious complications and early mortality. This approach is consistent with what is known about the pathophysiology of LCCN. Despite the ability of novel agents to induce rapid hematologic responses in most patients, the reduction in FLC during the first week remains critical to minimize ongoing renal injury and facilitate renal recovery. Early FLC control by HCO/MCO-HD may, therefore, complement, rather than replace, the effects of modern induction therapy. It is also noteworthy that early FLC responders likely represent a subgroup with inherently better treatment responses and overall survival. This observation highlights the difficulty of separating the effects of disease biology, treatment intensity, and HCO/MCO-HD. Larger studies with separate treatment strategies are needed to delineate the relative contributions of HCO/MCO-HD, anti-myeloma therapy with novel agents, and underlying myeloma biology.

## 5. Conclusions

There is currently limited data on the combined use of induction anti-myeloma therapy and extracorporeal FLC removal in patients with AKI and newly diagnosed MM. In this setting, our findings show that MCO-HD achieves kapa FLC reductions comparable to the lambda FLC reductions achieved with HCO-HD, suggesting that MCO-HD can be used in place of HCO-HD in kapa-restricted myeloma. Nonetheless, the necessity of employing an extracorporeal approach in patients receiving anti-myeloma treatment has not been established.

In this retrospective cohort, HCO/MCO-HD treatment alongside anti-myeloma therapy led to sustained FLC reductions and a possible contribution to renal recovery, although the separate efficacy of the two approaches cannot be determined. Prospective randomized studies are required to define the role of time-limited HCO/MCO-HD in the era of modern induction therapy.

## Figures and Tables

**Table 1:** Patient characteristics. RISS—Revised International Staging System; dUP—daily urine protein; eGFR—estimated glomerular filtration rate; creat—creatinine; LDH—lactate dehydrogenase; FLC—free light chain; Dg—date of diagnosis; Th—date of therapy; HCO/MCO-HD—high- and medium-cut-off membrane hemodialysis.

N = 33	
Median age (range)	66 (41–88)
Age > 70 N (%)	12 (36%)
Male N (%)	18 (55%)
Female N (%)	15 (45%)
Myeloma type N (%)	
Bence Jones protein	21 (64%)
IgG	9 (27%)
IgA	3 (9%)
Cytogenetic high-risk N (%)	11 (33%)
RISS N (%)	
1	3 (9%)
2	9 (27%)
3	14 (42%)
dUP, median (Q1–Q3)	3.6 (1.9–8.3) g/day
Creatinine, median (Q1–Q3)	471 (360–725)
eGFR (IQR)	8 (5–12)
eGFR < 15 mL/min/1.73 m^2^ N (%)	27 (82%)
Albumin, mean (SD)	37.1 (±7.5) g/L
Urine protein/creat, median (Q1–Q3)	423 (247–1030) g/mol
Urine albumin/creat, median (Q1–Q3)	25 (13–40) g/mol
LDH, median (Q1–Q3)	3.9 (3.0–4.5)
LDH, above reference N (%)	11 (33%)
FLC at diagnosis, median (Q1–Q3)	9630 (5220–16,000) mg/L
FLC ratio at diagnosis, median (Q1–Q3)	560 (250–1100)
Treatment	
Bortezomib-based triplet	17 (52%)
Bortezomib doublet	10 (30%)
Daratumumab quadruplet	4 (12%)
Daratumumab triplet	2 (6%)
Days from Dg to Th, median (Q1–Q3)	0 (0–2)
Days from Dg to HD, median (Q1–Q3)	0 (0–0)
HCO/MCO-HD procedures, median (Q1–Q3)	4 (3–6)

## Data Availability

The datasets generated during and/or analyzed during the current study are part of an ongoing project. Partial datasets will be available from the corresponding author upon reasonable request.

## Abbreviations

The following abbreviations are used in this manuscript:

AKI	Acute kidney injury
CR	Complete response
dUP	Daily urine protein
eGFR	Estimated glomerular filtration rate
FLCs	Free light chains
HCO-HD	High-cut-off hemodialysis
HSCT	Hematopoietic stem cell transplantation
LDH	Lactate dehydrogenase
LCCN	Light-chain cast nephropathy
MCO--HD	Medium-cut-off hemodialysis
MM	Multiple myeloma
OR	Overall response
PR	Partial response
RISS	Revised International Staging System
VGPR	Very good partial response
